# Supplementary material for: Are carbon emissions trading and green financial instruments synergistic? -Comprehensive quantitative research based on content analysis
Source: PLoS One. 2024 Mar 7;19(3):e0298601. doi: 10.1371/journal.pone.0298601 (PMC10919601; doi:10.1371/journal.pone.0298601)
Supplement: S1 Data — (ZIP) [file pone.0298601.s001.zip › Data/地级市Z_F工作报告.zip!/╝¬┴╓/╦╔╘¡╩╨/└┤╫╘╣1⁄2╓┌║┼í╛┬φ┐╦╩2╛▌═°í┐_2011.docx]

各位代表：

　　现在，我代表市人民政府，向大会作工作报告，请各位代表连同《松原市国民经济和社会发展第十二个五年规划纲要（草案）》（以下简称《纲要（草案）》）一并审议，并请市政协各位委员提出意见。

　　一、“十一五”经济和社会发展回顾

　　“十一五”期间，在省委、省政府和市委的正确领导下，我们团结和带领全市各族人民，励精图治，开拓进取，有效应对金融危机和历史特大洪涝灾害等一系列严峻考验，全面完成了“十一五”规划目标，全市经济快速发展、社会和谐稳定、人民安居乐业，各项事业取得了令人瞩目的巨大成就。

　　（一）经济发展实现新跨越。全市主要经济指标实现翻番增长，经济总量居全省第三位，入选跨国公司眼中最具投资潜力的中国城市20强，荣获建国60周年中国城市发展代表奖。2010年，全市地区生产总值预计（下同）实现1100亿元，人均地区生产总值突破38000元，分别是2005年的3倍和2.9倍；县域经济占全市经济比重突破70%。全口径财政收入完成75.1亿元，地方级财政收入完成31亿元，分别是2005年的2.2倍和2.8倍。社会消费品零售总额实现330亿元，是2005年的3.1倍。“十一五”期间，累计完成固定资产投资2960亿元，是“十五”时期的6倍。

　　（二）发展质量得到新提升。深入实施“工业立市”战略，扎实开展大项目建设“突破年”、“攻坚年”、“创新年”和“效益年”活动，274个投资亿元以上工业项目建成投产。吉林油田油气产量当量达到750万吨，大唐长山热电厂60万千瓦1号机组通过国家核准，长化集团、松原石化等一批重点企业扩能升级，嘉吉生化、吉安生化、正望油脂等企业成功实现重组。规模以上工业企业发展到611户，比2005年增加454户；规模以上工业增加值达到470亿元，是2005年的2.6倍。农业和农村经济发展步伐加快，哈达山水利枢纽、松原灌区和西部土地整理等重大农业基础设施项目建设稳步推进，全市粮食生产能力突破700万吨。规模以上农业产业化龙头企业发展到600户，比2005年翻一番。牧业大市建设成果显著，牧业产值占农业产值比重超过40%。服务业发展全面提速、增势强劲，金钻商圈、云天化600万吨物流等一批项目投入运营。“查干湖冬捕”入选“吉林八景”。2010年，全市旅游综合收入突破28亿元，是2005年的6倍。第三产业增加值达到370亿元，是2005年的4.6倍，增速连续四年在全省保持领先。三次产业比重由2005年的23.7:54.1:22.2调整到16.4:50:33.6。万元GDP能耗由1.33吨标煤降低到1.09吨标煤。

　　（三）城乡建设彰显新风貌。完成了《松原市城市总体规划》修编和滨江新区等专项规划，中心城区建成区面积由2005年的35平方公里扩大到56平方公里。“五城”联创取得重要成果，成功创建国家园林城市。松原大桥、康宁街公铁立交桥、城区街路、污水和垃圾处理、供热、供气、供水以及城市绿化、美化、亮化等一批民生工程陆续完成，城市承载功能明显增强。大广高速松原段、珲乌高速松原段建成通车，绕城高速、松陶铁路、松原港等重大交通基础设施项目建设全面展开，立体化大交通格局基本形成。累计建成公路8062公里，域内高速公路竣工里程居全省首位。县城、小城镇建设步伐加快，新农村建设扎实推进，城乡面貌焕然一新。城镇化率突破40%。“十一五”期间，我市还开通了进京列车、获准命名“松原星”，城市知名度和影响力全面提升。

　　（四）改革创新迈出新步伐。政府机构改革、粮食购销企业改革、国有企业改制并轨顺利完成，集体林权制度主体改革和中小学教师职称制度改革试点扎实推进。成功引进16家域外金融机构，成立11家小额贷款公司，各类担保公司发展到6户。东北石油技术服务公司在纽约交易所上市。民营经济增加值占全市GDP比重达到37%。发展中国驰名商标1件、国家地理标志商标4件、吉林省著名商标29件。“十一五”期间，全市累计完成招商引资1551.6亿元，实际利用外资4.3亿美元，完成进出口总额5.1亿美元，分别是“十五”时期的8.4倍、4.5倍和5倍。

　　（五）社会事业取得新成效。科技事业稳步推进，获省级科技成果奖20项、授权专利100件，顺利实施30个重大科技专项。市职业技术学院一期、滨江中学新建和农村校舍改造等一批教育基础设施项目建设全面推进。城乡医疗卫生条件明显改善，农民参合率稳步提升，疾病预防控制成效明显。文化艺术精品不断涌现，大型满族新城戏《洪皓》、神话吉剧《大布苏》等剧目获多项国家级大奖。前郭县民族歌舞团荣获全国“双服”先进集体称号。竞技体育水平明显提高，群众性体育活动广泛开展，圆满完成北京奥运会火炬接力松原传递活动。市群众艺术馆、博物馆、图书馆、体育馆、规划展览馆等项目建设接近尾声。市广电大楼和演播大厅等基础设施建设全面完成，广播电视采编播质量不断提高。认真落实计划生育基本国策，人口控制能力不断增强，第六次全国人口普查全面启动。市老年大学教学办公用房得到解决。“双拥”共建成果显著，征兵工作、国防动员、民兵预备役部队非战争军事行动能力建设受到国防部、沈阳军区和省军区的表彰。妇女儿童、老龄、民族、宗教、外事、档案、地方志、气象、地震、红十字等社会事业全面发展。

　　（六）民生改善达到新水平。规范和增加了机关事业单位工作人员津贴补贴。城镇居民人均可支配收入和农民人均纯收入分别达到16800元和5660元，分别是2005年的1.9倍和1.7倍。城镇累计新增就业23.8万人,城镇登记失业率控制在3.5%以内。社会保障体系日益完善，养老、失业、医疗、工伤和生育保险等保障水平全面提升。新型农村社会养老保险试点顺利实施，城乡低保实现动态管理下的应保尽保。社会救助力度不断加大。市社会福利服务中心、救助站、社会精神病院、儿童福利院陆续建成。残疾人抢救性康复工作步入全国前列。改造棚户区500万平方米，改造农村泥草房14.5万户。解决了近60万农村人口安全饮水问题。安全生产水平明显提高。环境保护力度不断加大，松花江规划项目和“十一五”减排目标全面完成，生态建设取得重要成果。对口援建四川黑水地震灾区任务圆满完成。

　　（七）民主法制建设取得新突破。主动接受市人大及其常委会的法律监督、工作监督和市政协的民主监督，自觉接受社会监督。注重加强同各民主党派、工商联、无党派人士及人民团体的沟通联系。五年累计办理市人大代表建议和政协委员提案740件，办复率达到100%，满意率逐年提升。依法行政、行政复议和行政执法监督工作不断加强，审计监督、行政监察和反腐倡廉力度加大，政行风和软环境建设扎实推进。“法治松原”、“平安松原”建设和“五五”普法成效明显。以严打整治为重点的社会治安综合治理强力推进，打黑除恶取得重要成果，广大人民群众安全感普遍提升。

　　2010年是实施“十一五”规划的最后一年，也是松原经济强力攻坚、克难奋进的一年。面对国际金融危机的持续影响和历史特大洪涝灾害的严峻考验，全市经济回升向好，完成了调整后的发展指标。全市地区生产总值同比（下同）增长15%，全口径财政收入和地方级财政收入分别增长1.1%和19.9%，全社会固定资产投资增长33%，社会消费品零售总额增长19.5%，城镇居民人均可支配收入和农民人均纯收入分别增长9.9%和10.4%。全市城乡居民生活水平明显提高，社会保障体系不断完善，精神文明和民主法制建设硕果累累，各项社会事业都取得了重要的发展成果。

　　各位代表，过去的五年，是松原发展极不平凡的五年。回顾这段历程，给了我们很多重要启示，主要是：必须坚持加快发展不动摇，千方百计扩大总量，只有把总量做大，才能在激烈的竞争中站稳脚跟；必须坚持结构调整不动摇，积极推动三次产业协调发展，只有把结构问题解决好，才能从容应对各种困难和风险的考验；必须坚持加快转变经济发展方式不动摇，努力做到快中求好、优质高效，只有全面提升经济运行质量，才能使松原的发展更加稳健；必须坚持强化城市建设管理不动摇，花大力气“筑巢引凤”，只有不断提升城市承载功能，才能更好地发挥辐射带动和引领作用；必须坚持改善民生不动摇，想方设法让人民群众过上更加幸福的美好生活，只有把群众关心的事情办好，才能凝聚推进发展的强大动力；必须坚持党的领导不动摇，在市委统一领导下，全市上下一条心、一盘棋，只有上上下下齐心协力，才能不断开创松原发展的美好明天！

　　过去的五年，是松原建市以来经济发展最快、社会效益最好、城乡面貌变化最大、人民得到实惠最多的五年。松原的发展进步，得益于省委、省政府和市委的正确领导，得益于市人大、市政协及各民主党派的监督支持，得益于全市广大干部群众和社会各界的团结奋斗，得益于离退休老领导、老干部的热情关注。在此，我代表市人民政府，向全市人大代表、政协委员、各民主党派、工商联、无党派人士、各人民团体和社会各界人士，向各中省直单位、驻松部队、武警官兵和公安干警，向全市各条战线干部职工、广大人民群众和关心支持松原发展的各界朋友，表示崇高的敬意和衷心的感谢！

　　各位代表，在看到成绩的同时，我们也清醒地认识到，我市经济社会发展还存在一些不容忽视的问题，主要是：经济总量不大，加快发展的基础不够牢固；产业结构不优，接续产业发展较慢；经济运行质量不高，企业市场竞争能力较弱；农业现代化程度不高，农民收入增长相对缓慢；一些民生问题有待解决，有些不稳定因素依然存在；行政效能与发展要求存在差距，政府自身建设还有待加强。对此,必须保持清醒头脑，采取更加有力的措施，认真加以解决。

　　二、“十二五”发展的基本思路和目标任务

　　“十二五”时期，是松原加快发展的重要战略机遇期。站在新的历史起点，《纲要（草案）》准确把握松原经济社会发展的阶段性特征，提出了“十二五”时期全市经济社会发展的总体思路，即：高举中国特色社会主义伟大旗帜，以邓小平理论和“三个代表”重要思想为指导，深入贯彻落实科学发展观，紧紧抓住科学发展这个主题、加快转变经济发展方式这条主线，统筹推进工业化、城镇化和农业现代化，强化投资拉动、项目带动和创新驱动，大力保障和改善民生，加快建设实力松原、魅力松原、文化松原、和谐松原、生态松原，推进资源型城市全面转型，努力开创经济总量更大、产业结构更优、城乡面貌更美、人民生活更加幸福、经济社会全面协调可持续发展的崭新局面，全力打造东北地区现代化区域中心城市，为提前实现全面建设小康社会目标奠定坚实基础。按照这个思路，“十二五”时期，要围绕加快发展、调整结构、改善民生，重点建设3大国家级产业基地，发展10大重点产业，实施12大发展计划，建设30个重大项目，提升松原在东北地区乃至全国的战略地位，实现发展新跨越。现在，《纲要（草案）》已经印发给大家，这里我仅就“十二五”发展目标和主要任务向大会报告。

　　（一）推动经济加快发展，快速壮大经济总量。从东北内陆欠发达资源型城市的实际出发，加快经济发展速度，做大经济总量。到2015年，全市地区生产总值达到3000亿元，年均增长22.2%。全口径财政收入和地方级财政收入分别达到300亿元和120亿元，年均分别增长32%。经济总量稳居全省第三位，进入全省经济规模“第一方阵”。以大投资拉动大发展。坚持把投资作为推动经济发展的重要动力，优化投资结构，提高投资质量，五年累计完成固定资产投资6800亿元，年均增长25%以上。以大项目带动大发展。突出抓好事关全局的30个重大项目，重点加快百亿级、千亿级产业建设。围绕国家产业和政策导向，挖掘松原的资源、区位等比较优势，谋划储备一批重大项目。以大创新驱动大发展。深入实施科教兴市战略和人才强市战略，加快发展创新型经济，建设创新型城市，增强产业核心竞争力。抢抓先行先试机遇，争取建设国、省级相关改革试验区。以大园区支撑大发展。加速园区提档升级，推动松原经济技术开发区、松原农业高新技术开发区向国家级开发区升级，查干湖旅游经济开发区争创国家级生态旅游示范区和文化产业示范基地，争取郭尔罗斯等一批省级工业集中区晋升为省级开发区。尽快做大松原石化产业园区，积极发展农机、食品医药、化肥等特色产业园，把园区经济发展成推动经济增长的重要引擎。以大开放促进大发展。扩大对外宣传，展示松原形象，积极参与长吉图经济区产业分工，实现联动发展。着力引进战略投资者，五年完成招商引资4000亿元以上。

　　（二）加快转变发展方式，促进经济结构优化升级。围绕加快石油资源型城市全面转型，把结构调整作为转变经济发展方式的主攻方向，加快发展战略性新兴产业，促进资源在与高端产业融合中实现效益最大化。加强资源环境管理保护，促进资源节约利用，发展循环经济，争取国家和省碳排放交易试点、低碳经济试点。万元GDP能耗比“十一五”末降低14%，主要污染物排放达到国家要求。优化三次产业结构。围绕构筑“四四二”产业格局，做大做强油气开采、化工、农畜产品加工、新能源4个主导产业，培育壮大旅游休闲、新材料、生物医药、文化创意4个战略性新兴产业，巩固提高装备制造、商贸物流2个传统优势产业，初步形成具有较强竞争优势的现代产业体系。到2015年，三次产业比重调整到10∶53∶37。优化工业内部结构。围绕打造国家重要的油气生产基地，全力支持吉林油田等重点企业扩能上产。按照资源可为、产业可续、产品可补的发展方向，培育壮大接续产业，提高油气资源就地转化率，推进生物质资源深度开发，延长农畜产品加工链条，加快发展地方工业。围绕优化产品结构，提高工业产品科技含量，打造一批具有市场竞争力的特色、优势品牌。优化农业产业结构。围绕农业与工业、服务业融合发展，培育循环农业、节水型农业、特色农业和休闲农业，争取建设国际食品安全区，促进农畜资源精深加工转化，提高农业综合效益。优化服务业结构。把服务业作为产业结构优化升级的战略重点，突出发展金融、物流、文化创意等生产性服务业，加快发展商贸、旅游、房地产、休闲娱乐等生活性服务业，向物流枢纽之都、商贸聚集之城、北国旅游之乡跨越。社会消费品零售总额达到900亿元，服务业增加值达到1110亿元，年均分别增长22.2%和24.6%。优化所有制结构。促进民营经济腾飞和中小企业成长，在配套投资、贴息补助、奖励资金等方面对民营企业给予更多支持，发展壮大一批骨干民营企业。民营经济增加值占GDP比重达到50%。优化发展动力结构。扩大消费需求，培育消费热点，鼓励扩大出口，促进经济发展由主要依靠投资拉动向消费、投资、出口多方面拉动转变。

　　（三）紧紧抓住“三化”统筹，构建松原特色发展格局。坚持在全国、全省的大视野下，统筹推进城镇化、工业化与农业现代化，实现“三化”联动发展。推进城镇化建设率先突破。立足建设“百年松原”，坚持高投入、高强度、高标准，扩展城市发展空间，构建“1139”城镇体系，构筑中心城市、节点城市、县城和小城镇梯次发展、“四位一体”的城镇化发展新格局。到2015年，城镇化率达到55%左右。坚持用规划引领城市发展，中心城区建成区面积达到80平方公里。规划新建江南、哈达山两个行政区，建设郭尔罗斯新城区，增强中心城市辐射带动能力。深入实施沿江开发战略，着力打造滨江新区，建设北方生态宜居精品城市。加快“五城”创建步伐，全面实现“五城”创建目标。强化城市功能配套，全面推进“五铁”、“四公”、“三港”等重大基础设施项目建设，完成“气化松原”建设任务，进一步增强城市综合承载能力。推动城镇内涵式发展，突出打造扶余中等城市，重点建设乾安、长岭、长山3个组团小城市，加快培育太平川等9个特色小城镇，增强城镇集聚产业和人口的能力。研究制定户籍、就业等配套措施，健全社会保障体系，加快城乡一体化进程。坚持走新型工业化道路。围绕产业高级化、技术高端化、产品高质化，建设一批国家级、东北级产业基地，做大做强特色产业。支持企业技术改造和自主创新，发展一批具有核心竞争力的大中型企业。到2015年，全市规模以上工业企业达到1100户，力争新上市企业6户，实现规模以上工业增加值1380亿元，年均增长24%。加快农业现代化建设步伐。围绕现代农业推进计划，加大农业科技创新力度，推动传统农业大市向现代农业强市迈进。加快建设哈达山水利枢纽、松原灌区和西部土地整理3大工程，突出抓好农田水利基础设施建设，加快建设标准基本农田，提高抵御各类自然灾害的能力。到2015年，全市粮食生产能力达到1000万吨水平。推进畜牧业扩量升级，形成支撑农村经济的“半壁江山”。围绕建设10大优质农畜产品生产基地和6大现代农业产业园区，引进大型农业产业化龙头企业。建立规范有序的土地经营权流转机制，促进土地集约经营。深入推进新农村建设，大力发展集体经济，多渠道促进农民增收。继续推进县域突破，实现县域综合经济实力在全省位次前移。到2015年，第一产业增加值实现300亿元，年均增长10.8%。

　　（四）千方百计改善民生，不断提高人民生活质量。把改善民生作为政府工作的重中之重，将更多的公共资源投向民生领域，推进基本公共服务均等化，让广大人民群众生活得更有保障、更有质量、更有尊严。大力实施富民工程。广泛开展全民创业带就业系列活动，实现城乡就业更加充分。到2015年，城镇新增就业25万人，城镇登记失业率控制在4.5%以内。努力增加城乡居民工资性、财产性、经营性收入，城镇居民人均可支配收入和农民人均纯收入分别达到39800元和12000元，年均分别增长18.8%和16.2%，全市人均收入水平位居全省前列。各项社会保障、救助体系更加完善，三年内实现城镇居民5项社会保险全覆盖；五年内实现新型农村社会养老保险全覆盖。加快发展社会福利、慈善和残疾人事业，全面提高城乡困难群体救助能力。推进安居工程和暖房子工程，逐步建立多层次住房保障体系。统筹发展社会事业。加快提升科技创新能力，科技进步贡献率达到65%。坚持教育优先发展，逐步加大教育投入，全面改善办学条件，不断提高基础教育质量，大力发展民办教育、职业教育和高等教育。健全城乡基本医疗服务体系。加快发展文化事业，提升文化软实力。推进市文化活动中心、数字电影城等文化基础设施建设，完成公共体育基础设施建设“五个一”工程，广泛开展全民健身活动。继续稳定低生育水平，不断提高人口素质。稳步推进其他各项社会事业。

　　（五）不断提升社会管理水平，努力构建和谐松原。适应社会结构新变化和开放性、流动性增强的新趋势，积极推进社会管理创新，使广大人民群众安居乐业。加快探索城乡社区建设，凝聚社会管理服务合力。深入开展法制宣传教育，维护社会公平正义。支持驻松部队和预备役建设，争创国家双拥模范城。认真落实党的民族宗教政策，促进民族团结进步。加强和改进信访工作，着力解决信访难题。扎实开展“平安松原”创建活动，加强社会治安综合治理。提高各类突发事件管理和应急响应能力，坚决防止重特大安全事故发生。不断强化政府效能建设，全面提升机关作风和政行风建设水平，打造更加宽松和谐的经济发展软环境。

　　三、2011年主要工作

　　2011年是“十二五”开局之年，也是继往开来、奋进跨越的关键一年。按照“十二五”规划目标，今年政府工作要全面贯彻中央十七届五中全会和省、市委全会精神，进一步突出加快发展、调整结构、改善民生这三个重点，努力实现经济总量新扩张、产业结构新优化、城乡面貌新改观、人民生活新改善，确保实现“十二五”发展开门红。

　　今年全市经济社会发展主要预期目标是：地区生产总值达到1360亿元，增长23.６%；全口径财政收入实现100亿元，地方级财政收入实现41.5亿元，均增长33.3%；全社会固定资产投资完成1200亿元，其中500万元以上项目固定资产投资完成819亿元，增长30%；社会消费品零售总额完成390亿元，增长19.3%；城镇居民人均可支配收入突破19960元，农民人均纯收入达到6580元，分别增长18.8%和16.2%；城镇登记失业率控制在4%以内；万元GDP能耗下降2.8%。实现上述目标，重点做好以下六个方面工作：

　　（一）以项目建设为支撑，着力做大经济总量。紧紧围绕总量扩张，扩大投资规模，全力推进重点项目建设。开工建设一批重大产业项目。每个县（区）和经济技术开发区建设亿元以上工业项目18个，其中5亿元以上工业项目3个。全市新开工建设中兴恒和二氧化碳基降解塑料、山东威高医疗器械加工、北车风机设备制造等亿元以上项目110个。实施一批重大交通和基础设施项目。开工建设内陆港，加快建设松原港、绕城高速、松陶铁路、哈大客专松原段，做好五常至科右中旗、大蒲柴河至科左中旗、嫩江至通辽高速公路松原段和长太白铁路、长白快铁松原段以及松原港疏港铁路、松乾铁路前期工作，力争完成松原查干湖机场项目前期工作。谋划一批重大储备项目。准确把握政策取向、产业导向和投资方向，突出基础设施建设、优势资源开发和节能减排等重点，切实搞好重大项目谋划，新储备亿元以上工业项目150个，其中5亿元以上项目30个。提高招商引资实效性。坚持开展“六个走进”活动，围绕“四四二”产业发展走向，主攻央企、大型国企和上市公司等重点企业，争取在引进央企投资项目上率先突破。积极参加东博会、农博会等各类展会，展示松原特色，扩大交流合作。招商引资到位资金完成575亿元，增长15%。大力发展民营经济。制定土地、税收、融资、人才等支持政策，做实做强创业孵化基地。完成民营经济主营业务收入980亿元，增长30.7%。

　　（二）以扶优促强为核心，着力推动工业提速增效。全面整合科技、信息、人才、资金等发展要素，强力推进石油、天然气、二氧化碳、农畜产品、风力、生物质等优势资源转化。全市规模以上工业企业达到700户,完成规模以上工业增加值575亿元，增长22.3%。扶持壮大主导产业。全力推动吉林油田、中石化东北油气、松原石化和奥瑞海山农机等一批重点企业扩大产能，加快长化集团“45-80”扩产改造，推进扶余路口电厂、大唐松原热电厂60万千瓦城区热电联供和大唐长山热电厂60万千瓦2号机组项目前期工作。推进在建和建成项目尽快投产达效，支持嘉吉生化、来禾化工、吉林天原生化、天安生物制品等企业加快扩能改造，鼓励重点企业上市，加快培育大型工业企业。提升企业综合素质和竞争实力。围绕管理标准化、技术先进化和生产规模化，规范企业法人治理结构，鼓励企业引进先进技术，加大研发投入，培育一批知名企业和优势品牌。实施科技开发和技改项目100个，开发投产新产品40种，培育中国驰名商标1件、省级著名商标3件。积极培育和引进大型外贸企业，进出口总额达到1亿美元。提高开发区和园区承载能力。完善园区发展规划，提高园区综合配套能力。探索园区、开发区公司化、市场化运营机制，鼓励园区、开发区进行土地收储，石化、农机和化肥等特色园区建设实现突破，力争有1个省级工业集中区升级为省级开发区，2个市级工业园区升级为省级工业集中区。

　　（三）以发展现代农业为主线，着力壮大农村经济。推进公共财政向农村倾斜、基础设施向农村延伸、城市文明向农村辐射。第一产业实现增加值190亿元，增长5.6%。正常年景粮食产量达到700万吨以上。大力发展集约高效农业。推进农业产业化经营，狠抓农产品精深加工，延长产业链条，提高农产品附加值，新增规模以上农业产业化龙头企业100户，实现销售收入150亿元。促进农业集约化、规模化、标准化生产，重点建设前郭县王府站镇标准化农业产业示范区、乾安县农机化集约经营示范区和宁江区棚膜蔬菜生产基地，推进长岭县北正镇盐碱地开发整理，总结推广新的发展模式。健全农业社会化、市场化服务体系，发展各种形式的规模经营主体，启动建立县乡两级土地流转交易大厅，新发展农民专业合作社500个、农村经纪人5000人。稳步推动农业产业结构调整。突出发展特色农业，新建温室大棚1万亩，棚菜面积达到9万亩以上，蔬菜面积发展到67万亩，绿色、有机、无公害农产品发展到237万亩。加快建设现代牧业大市，抓好畜禽繁改、防疫和产业化、组织化、标准化生产经营。开工建设中以现代生态良种奶牛繁育示范园区。加强牧业担保融资服务，建设标准化规模养殖场（小区）200个，牧业产值增长15%。启动实施第二个十年绿化松原大地工程，推进城乡生态建设，加强森林资源管护，积极发展林业产业，探索集体林权制度配套改革。拓宽农民增收渠道，加快发展劳务经济，实现劳务收入25亿元。全力推进农业基础设施建设。加快粮食产能建设，哈达山坝区枢纽、防护区、输水干渠等主体工程全面完工，实现库区蓄水，5台机组并网发电。完成松原灌区立项，启动部分片区建设。加强耕地保护，扎实推进西部土地整理，扩大膜下滴灌等节水农业生产规模，建设稳产高产田780万亩，改造中低产田5万亩。完成新一代天气雷达系统建设，增强人工影响天气和防灾减灾能力。不断加快新农村建设步伐。推进“千村示范、万村提升”工程，实施新农村建设项目300个，新建农村社区50个。改造农村泥草房2万户，解决5万农村人口安全饮水问题。完成“四清四改”村300个，新建改建农村公路793.6公里。不断壮大县域经济实力。立足资源、区位等比较优势和产业基础，提升技术、人才、资本等要素集聚能力，壮大支柱产业，促进产业集群。县域经济总量突破1000亿元，增长25%以上。

　　（四）以提档升级为导向，着力寻求服务业发展新突破。推动服务业优化发展、创新发展，第三产业实现增加值480亿元，增长29.7%。促进旅游业快速发展。完成《松原市旅游业发展总体规划》修编。推进查干湖、哈达山、龙华、三江口、江心岛、泥林等景区景点建设。丰富旅游产品和线路，推进旅游商品开发。办好查干湖蒙古族民俗旅游节、查干湖冰雪渔猎文化旅游节等特色节庆活动，多渠道拓展区域性客源市场。力争完成哈达山旅游经济开发区申报组建。全年接待游客400万人次，实现旅游综合收入35亿元。促进商贸物流业提质升级。加快金钻广场、东镇国际城两大商圈和中东新天地购物广场建设，开工建设欧亚购物中心。培育和引进大型物流集团，建成物流公共应用交换平台，逐步形成覆盖县、乡、村三级物流网络。大力推进粮食物流园区建设，筹建单家农贸物流园区，规划建设农业高新技术开发区农副产品商贸物流园区。促进房地产业健康发展。合理调控房地产开发规模，落实住房消费政策，激活新建商品房、二手房和房屋租赁市场。全年开发房地产面积260万平方米。促进金融业稳步发展。推动金融和经济发展深度融合，鼓励域内金融机构结合松原实际创新金融产品，扩大信贷规模。吸引招商银行、光大银行等域外金融机构落户松原，争取新增5家小额贷款公司、2家村镇银行和1家股份制商业银行。扶持省信用担保投资公司松原分公司发展壮大，鼓励社会力量组建商业性、互助性和行业性担保公司。继续优化金融生态环境。加快发展经营性文化产业。突出地域特色，推动文化产业创新发展，鼓励发展文化创意、影视拍摄等新兴文化产业。办好伯都讷端午文化节等特色文化节庆活动。大力扶持市满族艺术剧院、前郭县民族歌舞团和马头琴乐团做大做强，全方位塑造具有鲜明特色的地域文化品牌。

　　（五）以“五城”联创为重点，着力加快城镇化步伐。巩固国家园林城创建成果，加快向国家森林城晋位。加快创建国家卫生城和全国文明城。争取建成全国优秀旅游目的地城市和国家环保模范城。优化城市空间布局。突出宜居、宜业、宜游特色，高起点、高标准、高水平编制中心城区示范样板区、城市色彩等3个专项规划。严格执行和完善《松原市城市总体规划》。市规划展览馆建成开馆。全面推进中心城市建设。加快滨江新区开发建设。组织实施“一坝、一渠、三桥、九路”等重点工程。开工建设壅水坝，改造无形泄支，开工建设瓦房街公铁立交桥和文化路立交桥，筹建松原五桥。完成铁西路、学府街、源江路等6条道路续建，开工建设前程大路、和平西路延伸段、伯都讷大街延伸段3条道路，改造10条巷路。加快建设江南公交指挥中心。完成松原星塔设计选址和镜湖改造。加快城市绿化提档升级。抓好城区南、西、北3个出口绿化工程，实施松原大路、伯都讷大街、滨江大道等主要街路绿化升级。以沿江两岸、桥梁、商圈、镜湖等为重点，突出抓好城市亮化、美化，彰显城市文化特色。新建江北富江苑热源厂，扩建江南热源厂，建设11座换热站。新建江南水厂，建设输配水管网45公里。完成“气化松原”年度建设任务。按照百年一遇标准，加快完善城市防洪工程。积极创新投融资模式，通过合作开发、发行债券和BOT、BT等形式，破解城市建设资金瓶颈。推进县城和小城镇建设。进一步完善县域城镇发展规划，加大城镇基础设施建设投入，高标准完成郭尔罗斯新城区和扶余中等城市年度建设任务。深化户籍制度改革，逐步解决进城务工人员就业、安居和社会保障等问题，促进农民向城镇有序转移。提高城市管理水平。整合城市管理资源，积极推进管理创新。坚持实行统一要求、统一标准、统一检查，确保各司其职，各负其责。引入市场机制，高标准装备城市环卫设施，加快推进“数字松原”建设，进一步提升城市品位。

　　（六）以改善民生为落脚点，着力促进社会和谐稳定。集中力量办好人民群众最关心、最期盼、最需要的好事、实事，让人民群众得到更多实惠。促进居民充分就业。加强职业培训和就业指导服务，努力提高劳动者就业技能和综合素质。新增城镇就业4.5万人，保持“零就业”家庭动态为零，转移农村剩余劳动力49万人次。提高社会保障水平。农村“五保”供养对象全部纳入保障范围。开展低收入家庭认定，规范低保管理。扩大新型农村社会养老保险覆盖面。推进医疗保险城乡一体化制度创新，实现市级统筹。提高医疗、失业和工伤保险统筹层次，参保率稳定在90%以上。完成市残疾人康复中心和托养中心一期工程建设。全面落实价格调控政策，保障城乡居民特别是中低收入群体基本生活不受影响。推进社会事业协调发展。继续加大教育投入，推进教育基础设施和标准化学校建设，加快实施中小学校舍安全工程，完成市特殊教育学校建设。突出发展学前教育、职业教育、民族教育，促进城乡各类教育均衡发展。适应经济社会发展需求，切实加强各类人才队伍建设，深入推进事业单位人事制度改革。建设名优酿酒葡萄生产等4个专利示范基地，搞好10个产学研项目对接。推进医药卫生体制改革，健全医疗卫生服务体系，严防重大传染性疾病。组织实施“健康快车”复明工程。推进市中心医院异地重建和市中西医结合医院、市生殖保健医院建设。完成7家社区卫生服务中心新建、改造任务。建设10个乡镇计生服务站，启动优生筛查和药物干预工程。繁荣发展文化事业，打造蒙满等民族特色文化精品，新创作剧本20个以上。筹建市文化活动中心，建成市群众艺术馆、博物馆、图书馆和体育馆。新建乡镇综合文化站、文化大院和农家书屋50个。大力开展全民健身活动，备战第十二届全国冬运会。全面推进广播电视“村村通”工程。继续推进棚户区改造、廉租房建设和暖房子工程，启动公租房建设。推广可再生能源建筑应用。加强地震监测，强化抗震设防和应急管理。推进人防组织指挥和综合防护能力建设。营造和谐稳定的发展环境。完善村务公开和民主管理机制，搞好“示范社区”建设试点。推进民族团结进步事业，依法管理宗教事务。启动“六五”普法，深入推进“法治松原”建设。规范市场秩序，清理整顿市场中介组织，强化食品药品安全监管。加强环保监管执法，推进环保基础设施建设，搞好饮用水源地保护，有效控制各类污染源。继续开展“双拥”共建，维护军政军民团结。狠抓信访维稳，解决信访突出问题。开展打黑除恶斗争，构筑坚强有力的社会治安防控体系。强化安全发展理念，全面落实安全生产主体责任，扎实开展隐患排查整治，坚决保障广大人民群众生命财产安全。

　　各位代表，完成上述发展任务，必须把加强政府自身建设摆在突出位置。在今后的工作中，一要严格依法行政，努力建设法治政府。坚持执行重大事项集体决策、专家咨询论证等制度，畅通行政复议渠道，推进政务公开，规范行政权力。主动接受人大及其常委会的法律监督、工作监督和政协的民主监督，认真执行人大决议决定，定期向人大、政协报告、通报工作。坚持面复制度，认真办理人大代表建议和政协委员提案。严格执行执法责任追究制，解决有法不依、执法不公等群众反映强烈的问题。二要提高行政效率，努力建设效能政府。深化行政审批制度改革，实现服务程序“链条化”，全面提高审批效率。健全绩效考核机制，严格规范奖惩，激发干事创业的积极性和创造性。强化公务员队伍建设，提高各级干部推动科学发展的能力。大力倡导务实作风，坚决反对形式主义、官僚主义，切实精简会议、压缩文件，逐步建立规范、高效、快捷的运转机制。三要倾力造福于民，努力建设民生政府。坚持把为人民群众谋福祉作为政府工作的最高追求，察民情、解民意、惜民力，尊重群众的知情权、参与权，尊重群众的合理诉求，关注困难群众的衣食住行、安危冷暖，把有限的财力向民生领域倾斜，多为老百姓做一些实实在在的事情。四要坚持从严治政，努力建设清廉政府。认真落实党风廉政建设责任制，扎实推动源头治腐。严格执行“一岗双责”，突出关键环节和重点领域，建立健全反腐倡廉长效机制。切实强化审计监督，搞好经济责任和专项审计。加强公共财政支出管理，坚决制止铺张浪费。不断强化政行风评议，搞好百名科（处）长评议活动。严肃查处违法违纪案件，严厉整治“不作为”和“乱作为”等突出问题，不断改善经济发展软环境。

　　各位代表，实现“十二五”和今年的发展目标责任重大、使命光荣。让我们在市委的正确领导下，紧紧团结和依靠全市各族人民，坚定信心，迎难而上，开拓奋进，为实现松原的科学发展、和谐发展、跨越发展而努力奋斗！
